# The fabrication of biomimetic biphasic CAN-PAC hydrogel with a seamless interfacial layer applied in osteochondral defect repair

**DOI:** 10.1038/boneres.2017.18

**Published:** 2017-07-04

**Authors:** Jinfeng Liao, Taoran Tian, Sirong Shi, Xueping Xie, Quanquan Ma, Guo Li, Yunfeng Lin

**Affiliations:** 1 State Key Laboratory of Oral Diseases, National Clinical Research Center for Oral Diseases, West China Hospital of Stomatology, Sichuan University, Chengdu, China

## Abstract

Cartilage tissue engineering based on biomimetic scaffolds has become a rapidly developing strategy for repairing cartilage defects. In this study, a biphasic CAN-PAC hydrogel for osteochondral defect (OCD) regeneration was fabricated based on the density difference between the two layers *via* a thermally reactive, rapid cross-linking method. The upper hydrogel was cross-linked by CSMA and NIPAm, and the lower hydrogel was composed of PECDA, AAm and PEGDA. The interface between the two layers was first grafted by the physical cross-linking of calcium gluconate and alginate, followed by the chemical cross-linking of the carbon-carbon double bonds in the other components. The pore sizes of the upper and lower hydrogels were ~187.4 and ~112.6 μm, respectively. The moduli of the upper and lower hydrogels were ~0.065 and ~0.261 MPa. This prepared bilayer hydrogel exhibited the characteristics of mimetic composition, mimetic structure and mimetic stiffness, which provided a microenvironment for sustaining cell attachment and viability. Meanwhile, the biodegradability and biocompatibility of the CAN-PAC hydrogel were examined *in vivo*. Furthermore, an osteochondral defect model was developed in rabbits, and the bilayer hydrogels were implanted into the defect. The regenerated tissues in the bilayer hydrogel group exhibited new translucent cartilage and repaired subchondral bone, indicating that the hydrogel can enhance the repair of osteochondral defects.

## Introduction

Articular cartilage is a tissue without blood vessels, lymphatic vessels or neural tubes. Therefore, it possesses a limited ability to heal itself when damaged.^1,2^ There are generally three models of cartilage defects based on the depth of damage: partial-thickness defects, full-thickness defects and osteochondral defects. Specifically, osteochondral defects are involved in injuries to the whole region of subchondral bone. The subchondral bone plays an important role in supplying support for the overlying articular cartilage.^3,4,5^ Osteochondral defects without treatment may lead to the deterioration of joint function. Thus, it is very important to repair osteochondral defects to prevent joint destruction.

Recently, tissue engineering has emerged as a critical method for the repair of injured tissues. Tissue engineering involves the design and fabrication of a scaffold that possesses similar structural and functional characteristics to those of the original tissue and that provides an appropriate microenvironment for regulating cell attachment, growth and integration. Thus, the properties of the scaffold, including pore size,^6,7^ porosity,^8,9^ substrate stiffness,^10,11,12^ and orientation,^13,14^ are known to be key determinants. For example, an evaluation of a gradient stiffness hydrogel for cell culture demonstrated that scaffolds with favorable stiffness ranges induced human bone marrow mesenchymal stem cells (hMSCs) to differentiate into specific cell types (20 KPa for nerve cells, 40 KPa for muscle cells, 80 KPa for chondrocytes, and 190 KPa for osteoblasts).^15^ However, the traditional cartilage tissue engineering approach was disappointing at regrowing new homogenous tissue, resulting in its failure at achieving widespread clinical effectiveness.^16^ For a “biomimetic” approach, the scaffold should possess the same properties as the host cartilage and subchondral bone when being used for osteochondral defect repair.

Therefore, stratified scaffolds have received more and more attention for assisting the growth of different types of cells in each layer and attempting to regenerate the corresponding tissues simultaneously. Biphasic scaffolds are superior for osteochondral tissue regeneration applications due to the fabrication of optimized microenvironments in different layers by changing the chemical, structural, and mechanical properties. The construction involved the fabrication of two separate scaffolds: one was designed to repair cartilage tissue, and the other was designed to repair bone tissue. These two layer scaffolds were subsequently fused together using biological sealants, glues or freeze drying.^17,18^ For example, Zhang *et al.* have fabricated a bilayer microporous scaffold with a collagen layer and poly-l-lactic acid nanofibers via the freeze-drying technique.^19^ Unfortunately, the stiffness of the scaffold was not addressed. Then, Steele *et al.* added aligned fiber membranes to improve the surface and mechanical properties of the particulate-templated scaffold.^20^ For further studies on osteochondral tissue engineering, researchers have spent a considerable amount of time on designing the scaffold. Bilayer silk/silk-nano CaP scaffolds were fabricated for simulating the cartilage layer and osteochondral layer.^21^ The *in vivo* osteochondral defect assessment revealed that glycosaminoglycan (GAG) and type II collagen (COL II) regeneration occurred in the silk layer and that vessels formed in the silk-nanoCaP layer.^21^ Moreover, growth factors were introduced into the biphasic scaffold. An osteochondral scaffold was prepared that included a transforming growth factor-β_1_-loaded chitosan-gelatin layer for cartilage formation and a BMP-2 loaded hydroxyapatite/chitosan-gelatin layer for bone formation.^22^ MSCs seeded in the bilayer scaffold presented high expression of the transforming growth factor-β_1_ protein in the upper layer and BMP-2 protein in the lower layer.^22^ Although these approaches had some interesting successes, these materials had some limitations, including a weak interface force between the two layers, poor cellular infiltration through the layers of the structure and the absence of the regeneration of the calcified cartilage region.^23^

**Figure 1:**
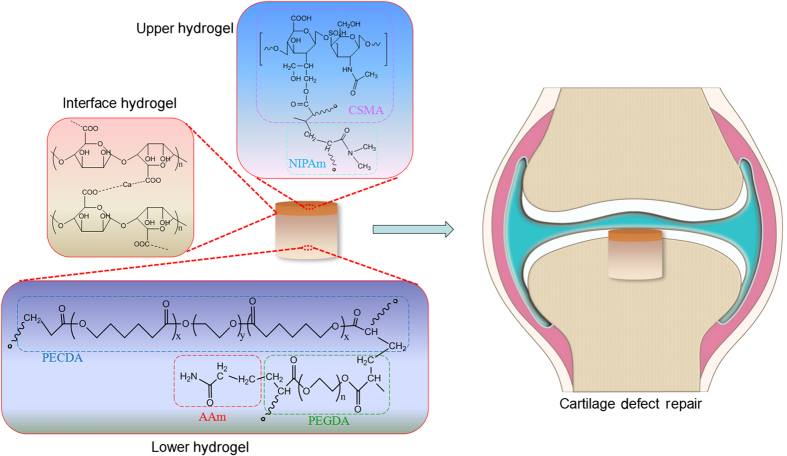
Scheme showing the designed biphasic CAN-PAC hydrogel applied in cartilage tissue engineering.

Among the materials available, hydrogels give hope to researchers for conquering these shortcomings because of their superior characteristics, including appropriate water uptake, efficient nutrient transportation, benign tractability for cell growth in a three-dimensional (3D) structure and effective integration with the original tissues after operation.^24,25,26^ In this study, we have developed a biphasic CAN-PAC hydrogel with a seamless interface that simulated the properties of osteochondral tissue. The bilayer hydrogel was fabricated using a one-pot heat-initiated free radical polymerization method with a density difference between the upper layer and lower layer, as shown in Figure 1. This hydrogel was designed specifically for the regeneration of osteochondral defects and prepared by a novel and simple method to create its distinct and seamless layers. The upper layer was grafted to the lower layer firstly by the physical cross-linking of calcium gluconate and alginate, followed by the chemical cross-linking of the carbon–carbon double bonds in the other components. The prepared CAN-PAC hydrogel thus demonstrated strong interfacial bonding and improved mechanical properties. This bilayer hydrogel exhibited the advantages of mimetic structure, mimetic composition, and mimetic stiffness, which gave us the opportunity to assess its ability for osteochondral defect repair. Cartilage and bone cells have good adhesion and proliferation on the biphasic hydrogel. Meanwhile, the biodegradability and biocompatibility of the biphasic hydrogel were examined *in vivo*. Furthermore, an osteochondral defect was developed in an animal model, and the effect of the biphasic hydrogel on the regeneration of the osteochondral injury was assessed.

## Materials and methods

### Macromer synthesis

The synthesis of methacrylated chondroitin sulfate (CSMA) followed the procedure from our previous study.^27^ The poly(*ε*-caprolactone)-poly(ethylene glycol)-poly(*ε*-caprolactone) (PCL-PEG-PCL) copolymer was synthesized by the ring-opening polymerization of ε-CL initiated with PEG and using Sn(Oct)_2_ as a catalyst. The obtained PCL-PEG-PCL was dissolved in CH_2_Cl_2_. Then, AC was added to the reaction mixture, which was refluxed reaction at 40 °C for 4 h. Meanwhile, a few drops of TEA were used to neutralize the formed acid. After the reaction, petroleum ether was added to precipitate the synthesized acryloyl chloride-poly(ε-caprolactone)-poly(ethylene glycol)-poly(ε-caprolactone)-acryloyl chloride (PECDA). The obtained product was dried under vacuum at 25 °C.

### Preparation of the biphasic CAN-PAC hydrogel

The CAN-PAC hydrogel was prepared by thermally initiated free radical polymerization. In brief, 250 mg of CSMA, 33 mg of NIPAm and 4.0 mg of BIS were dissolved in 1 mL of aqueous alginate solution (2%, w/v) with the assistance of ultrasonication (upper solution). At the same time, 300 mg of PECDA, 10 mg of AAm, 50 μL of PEGDA and 5.0 mg of BIS were dissolved in 1 mL calcium gluconate (3%, w/v) solution (lower solution). The initiator APS was added to the monomer solutions before polymerization. The upper solution (40 μL) was transferred into a 96-well plate. Then, 160 μL of the lower solution was added on top of the transferred upper solution. The plate was sealed and immersed in a water bath with the temperature set at 50 °C. The polymerization was allowed to run for 1 h. Then, the obtained hydrogel was immersed in excess distilled water to get rid of other residual impurities.

### Characterization of the CAN-PAC hydrogel

Fourier transform infrared (FTIR) spectra of the scaffolds and the macromonomers were analyzed on a Nicolet 200SXV spectrophotometer (Nicolet, Thermo Scientific, Waltham, MA, USA). The scaffold microarchitecture and porosity were analyzed using Micro-CT. Either the diameter or the height of the scaffolds was 5 mm. Specifically, the upper and lower layers were 2 and 3 mm in height, respectively. Three samples were employed for the microstructure assessment. The cross-section morphologies and mean pore sizes of the CAN-PAC scaffolds were examined using scanning electron microscope (SEM) (JSM-5900LV, JEOL, Tokyo, Japan). The swelling behavior of the CAN-PAC scaffolds was evaluated as follows. The dry weights of the scaffolds (Wd) were weighed after lyophilization. Then, at specific time points of immersion in PBS (pH=7.4) at 37 °C, the scaffolds were weighed with oil paper after the superficial water had been eliminated (Ws) (*n*=3). The percentage of water uptake (WU) was calculated as (Ws-Wd)/Wd)×100%. The compressive ability of the scaffolds, including the upper hydrogel, lower hydrogel and bilayer hydrogel (*n*=3) was measured in a universal testing machine (Instron 5565, Norwood, MA, USA). Cylinder-shaped hydrogel samples with a 16-mm radius and an 8-mm height were put on a test platform and tested in the wet state. The compression rate was set at 0.1 mm·min^−1^, and the test was finished once the hydrogels appeared to fracture. The stress–strain curves and elastic moduli of the hydrogels were obtained.

### Cell seeding and culture on hydrogels

The articular cartilage and osteoblast cells were obtained from the knee joints and skull of newborn mice, respectively. Then, the samples were cut into slices. Chondrocytes and osteoblast cells were digested from cartilage pieces (with 0.2 wt % collagenase II) and skull slices (with 0.2 wt % collagenase I), respectively. After complete digestion, the isolated cell suspension was filtered and centrifuged. Then, the obtained cells were cultured and incubated. The sterilized hydrogels were washed with PBS three times. Then, the hydrogels were immersed twice in culture supplements. After the cultured cells had grown to passage three, the cartilage cells and osteoblast cells were separately seeded onto the surface of the upper and lower hydrogels at a density of 1.0×10^5^ cells per hydrogel. After 12 h of seeding, the hydrogel/cell composites were moved into another plate to eliminate any unattached cells. At different time intervals (1 day, 4 days and 7 days), the increase in cell number was detected by the CCK-8 method. To obtain fluorescence photographs, the nuclei of the cells were stained with DAPI. Meanwhile, SEM was used to observe the adhesion of cells seeded on the hydrogels.

### Animal tests

Twelve healthy male Sprague-Dawley (SD) rats (~200 g) were employed to evaluate the degradation and biocompatibility of the CAN-PAC hydrogels. Eighteen healthy male New Zealand white rabbits (~2.0 kg) were used to investigate the cartilage repair ability of the hydrogels. The rats and rabbits were obtained from the Experimental Animals Center of Sichuan Province, China. The animal study was approved by the Animal Care and Use Ethics Committee at the State Key Laboratory of Oral Diseases, Sichuan University. The hydrogels used in the animal experiments were sterilized by irradiation with 25 kGy Cobalt-60 for 2 h at the Sichuan Academy of Agricultural Sciences.

### *In vivo* biocompatibility and degradability of CAN-PAC hydrogel

The *in vivo* biocompatibility of the bilayer hydrogel was evaluated after embedding hydrogels under the back skin of SD rats. At predetermined time intervals after implantation, animals were killed by overdose chloral hydrate. Photographs of the remaining hydrogels under the skin were taken. Then, the implanted materials and the surrounding tissues were removed carefully. The biocompatibility was evaluated by hematoxylin and eosin (H&E) staining.

### *In vivo* full-thickness cartilage defect repair

Eighteen white rabbits were used for the *in vivo* cartilage defect repair experiments. The animals were anesthetized by intramuscular injection of 30 mg/kg pentobarbital sodium prior to surgery. The region of operation was shaved and aseptically prepared for operation. An osteochondral defect (thickness: 4.0 mm; diameter: 5.0 mm) was fabricated through both the chondral and subchondral bone layers of the patellar groove in their left legs using an electric drill. The defects of the experiment group were treated with the bilayer hydrogel. The other group was treated with nothing as the control group. The wound was closed by suturing, and penicillin was given to each rabbit for 7 days following the operation. No animals were observed to be infected throughout the experimental period. The knee joint samples were collected at 6, 12, and 18 weeks after the operation. The samples were first assessed by a Micro-CT scanner (Micro-CT 80, Scanco, Switzerland). The scan parameters were set as follows: X-ray voltage=55 kV, X-ray current=90 μA, voxel resolution=20 μm and detector size=1 024×1 024. Then, the scan images were used to reconstruct 3D geometry in VGStudio MAX. For histological examination, the samples were stained with H&E, Saf-O, and toluidine blue. For biomechanical evaluation, samples from the two groups (18 weeks, *n*=3) and native osteochondral samples were tested. The compressive mechanical properties of the samples were tested on an Instron model test machine. A step displacement (0.20 mm strain) was applied, and the compressive force was measured until equilibrium was reached. The reduced modulus was calculated according to the biomechanical curves.

## RESULTS

### Synthesis and preparation of the CAN-PAC hydrogel

**Figure 2:**
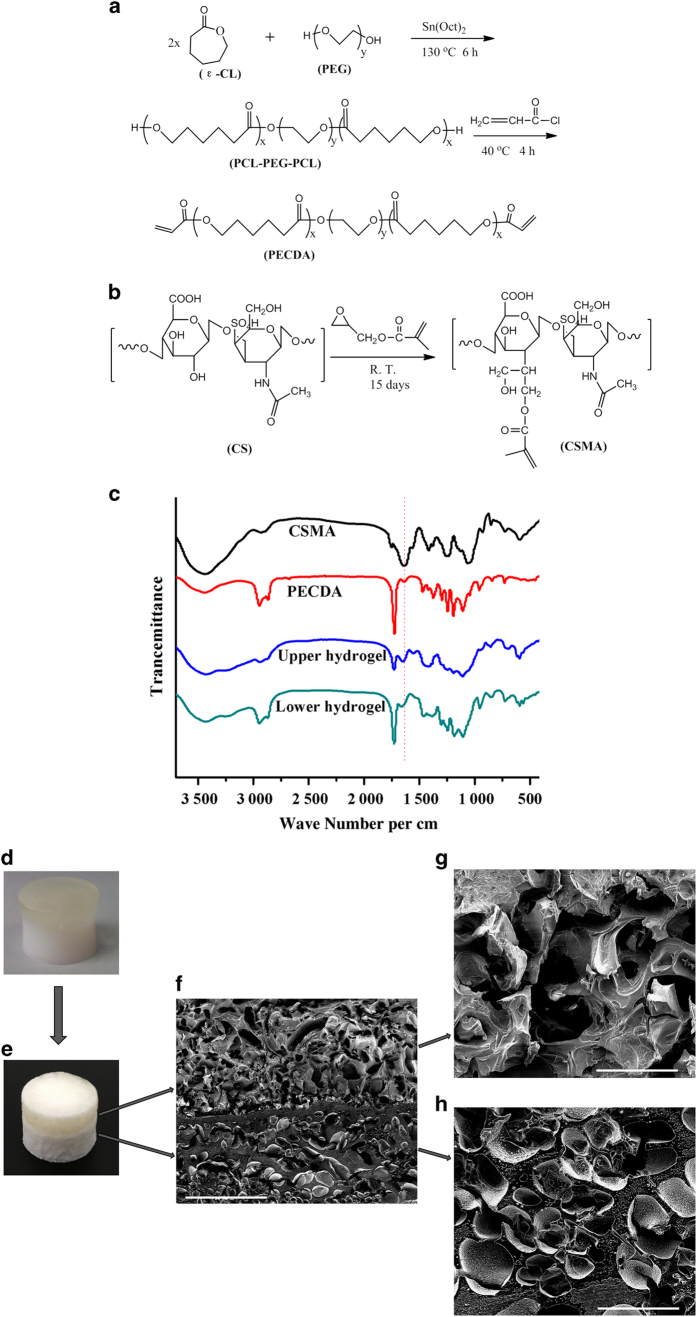
The preparation and characterization of CAN-PAC hydrogel. Scheme showing the synthesis of (**a**) PECDA and (**b**) CSMA. (**c**) FTIR spectra of CSMA, PECDA macromonomer, upper hydrogel and lower hydrogel. (**d**) The obtained CAN-PAC hydrogel. (**e**) The lyophilized CAN-PAC hydrogel. Cross-sectional SEM of the (**f**) biphasic CAN-PAC hydrogel (scale bar=1 mm), (**g**) upper hydrogel (scale bar=200 μm) and (**h**) lower hydrogel (scale bar=200 μm).

The PECDA copolymer and CSMA were synthesized as shown in Figure 2a and b. The FTIR spectra of CSMA, PECDA, upper hydrogel and lower hydrogel are presented in Figure 2c. After modification with GMA, CS displayed a new peak corresponding to C=O stretching (~1 736 per cm). The peaks at ~1 745 and ~1 122 per cm were attributed to ester vibrations in PECDA. The band at ~1 620 per cm was ascribed to C=C stretching and can be clearly observed in the FTIR spectra of CSMA and PECDA. This band obviously weakened in the upper and lower hydrogel spectra, which suggests that a majority of the carbon-carbon double bonds had been cross-linked to form carbon-carbon single bonds. CSMA and PECDA were cross-linked into the structure of the upper layer hydrogel and lower layer hydrogel, respectively.

### Characterization of the CAN-PAC hydrogel

To investigate the interior structure of biphasic hydrogel, SEM and Micro-CT were used to characterize it. Figure 2d shows a macroscopic image of the bilayer hydrogel. After lyophilization, the boundary of the CAN-PAC scaffold was not difficult to distinguish (Figure 2e). Furthermore, a cross-section of the lyophilized scaffold was observed via SEM. As shown in Figure 2f, the junction of the bilayer scaffold showed that the upper scaffold (chondral layer) was distinct from the lower scaffold (bone layer). Meanwhile, the two layers were firmly connected without any separated regions. This result proved that the upper scaffold and the lower scaffold could be integrated together with strong interfacial bonding. In addition, both the upper (Figure 2g) and lower (Figure 2h) scaffolds showed an interconnected porous structure. The upper scaffold had a larger pore size than the lower scaffold.

**Figure 3:**
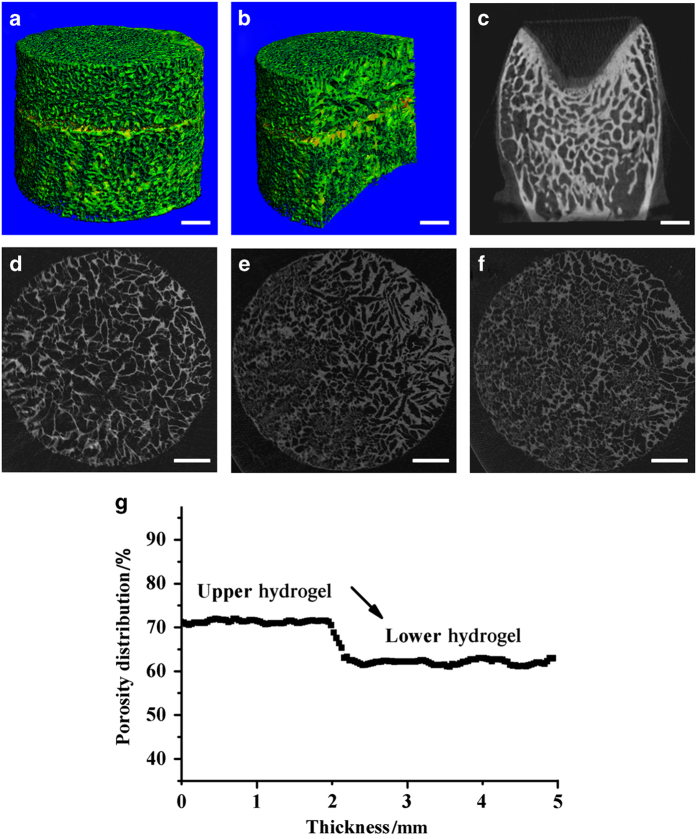
Morphology and quantitative analysis of CAN-PAC scaffold by Micro-CT. Micro-CT analysis using 3D reconstruction images of (**a**) the CAN-PAC scaffold and (**b**) a vertical section of the CAN-PAC scaffold. (**c**) 2D reconstruction of the cross-section of the articular joint from rabbits. 2D reconstruction of the (**d**) upper scaffold, (**e**) middle transition scaffold, and (**f**) lower scaffold (scale bar=1 mm). (**g**) Quantitative analysis of the porosity distribution in the whole freeze-dried biphasic CAN-PAC hydrogel.

For further investigation of the biphasic scaffold, we evaluated the scaffold by Micro-CT. 3D reconstruction images (Figure 3a), and vertical section images (Figure 3b) displayed two distinct phases in the bilayer scaffold. The two phases were well integrated with a seamless interface, as indicated by the yellow color in Figure 3a and b. In addition, it was easy to observe that the scaffold showed a porous structure, as was observed by SEM. Then, 2D images of the upper layer (Figure 3d), middle transition interface (Figure 3e) and lower layer (Figure 3f) were obtained. It is worth noting that the middle transition layer had the characteristics of both the upper and lower scaffolds. From the 2D reconstruction images of the scaffold and the articular joint from rabbits (Figure 3c), it was easy to see that the bilayer hydrogels showed a similar structure to that of cartilage and subchondral bone. Next, we used Micro-CT to quantitatively analyze the porosity distribution in the biphasic scaffold. The porosities of the upper scaffold and lower scaffold were ~71.5% and ~62.3%, respectively (Figure 3g). The porosity was homogeneously distributed throughout both layers. A sharp decrease in the porosity appeared in the transition interface region.

### Physicochemical properties of the CAN-PAC hydrogel

**Figure 4:**
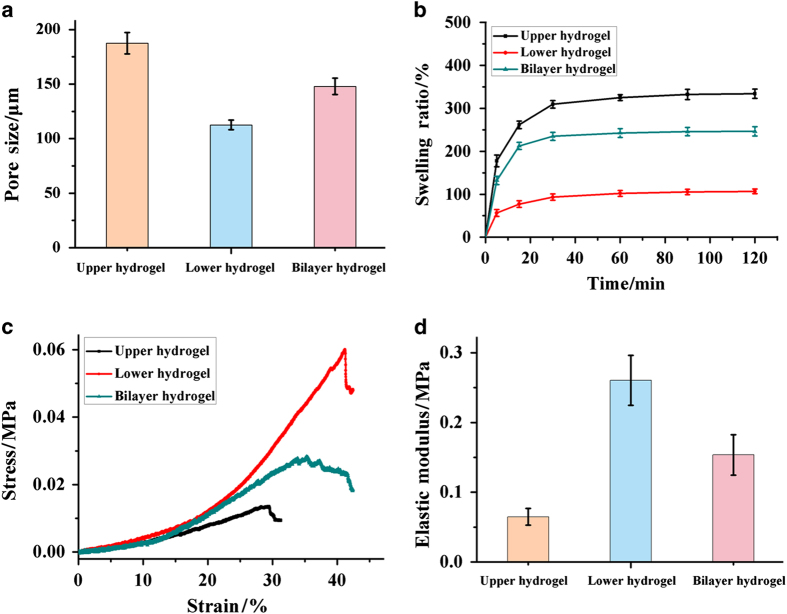
The physicochemical properties of CAN-PAC hydrogel. (**a**) The pore sizes, (**b**) swelling abilities, (**c**) stress curves and (**d**) elastic moduli of the upper hydrogel, lower hydrogel and biphasic hydrogel.

Cartilage tissue engineering requires not only an interconnected porous scaffold structure but also a reasonable pore size and distribution.^28^ The pore sizes of the upper hydrogel and lower hydrogel were ~187.4 and 112.6 μm, respectively (Figure 4a). In addition, the mean pore size of the whole hydrogel was ~147.9 μm, which would be beneficial for cell culture.

In the equilibrium swelling ratio test, the scaffolds were lyophilized before being tested. The samples absorbed PBS gradually and all reached swelling equilibrium within 30 min (Figure 4b). The swelling ratio of the upper hydrogel was higher than that of the lower hydrogel during the test period, which was related to the hydrophilicity of CSMA in the upper hydrogel. In addition, the whole hydrogel had a comprehensive swelling ability, which may help avoid problems such as scaffold protrusion during implantation. The variations in the equilibrium swelling ratio of the scaffold were consistent with their porosity. This might be explained by the fact that the upper hydrogel made from CSMA (as a main component) had a larger pore size and higher porosity, which provided it with more space to store water, resulting in the increase in water absorption.^27^

The mechanical properties of hydrogels are another main factor in cartilage defect repair because scaffolds implanted in a defect area are subject to dynamic loading. To measure the mechanical properties of the hydrogels (including the upper hydrogel, lower hydrogel, and bilayer hydrogel), we used the unconfined dynamic compression method. The stress–strain curves of the upper hydrogel and lower hydrogel from mechanical detection revealed that both layers had little stress at 0–15% strain of the hydrogels (Figure 4c). Then, they showed a linear increase of stress at 15%~29% strain for the upper hydrogel and 15%–41% strain for the lower hydrogel (Figure 4c). Above these strains, the upper and lower hydrogels were fractured by cracking. Meanwhile, the bilayer hydrogel tolerated a compression of 34% before they were fractured. It is interesting to note that the whole hydrogels were fractured gradually with compression between 34% and 41%. This may contribute to the seamless connection of the upper and lower hydrogels, which helps to scatter the force.

It was also necessary to evaluate the elastic moduli of the hydrogels. Stem cells can perceive and respond to the mechanical properties of an extracellular scaffold, which provides feedback to regulate their differentiation fate (0.020–0.080 MPa for cartilage, 0.110–0.190 MPa for osteoblasts).^10,15,29^ The wet state moduli of the upper hydrogel, lower hydrogel and bilayer hydrogel were ~0.065, ~0.261, and ~0.154 MPa, respectively (Figure 4d). This result indicated that the elastic moduli of upper hydrogel and lower hydrogel were the optimal stiffness for the growth of cartilage cells and osteoblast cells.

### Attachment, viability and proliferation of cells on CAN-PAC hydrogels

**Figure 5:**
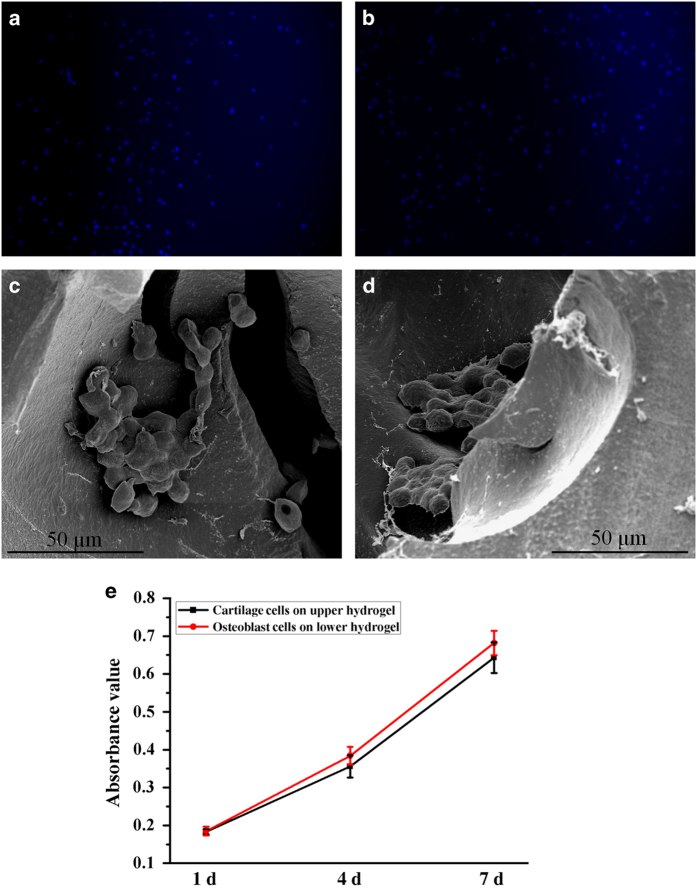
Cell viability on the biphasic hydrogel. (**a**, **b**) Fluorescence and (**c**, **d**) SEM images of cartilage cells on the upper hydrogel (**a**, **c**) and osteoblast cells on the lower hydrogel (**b**, **d**). (**e**) Cell proliferation on the CAN-PAC hydrogel measured using the CCK-8 assay.

To investigate the feasibility of the bilayer hydrogel as a biomimetic scaffold for cell growth, cartilage cells and osteoblast cells were separately grown on the upper hydrogel and lower hydrogel *in vitro*. After cells were seeded onto the surface of the scaffolds for 7 days, the cell nuclei were stained with DAPI to obtain fluorescence images (Figure 5a and b). Chondrocytes and osteoblast cells adhered and proliferated on each layer, suggesting good cell attachment and growth on the hydrogels. Meanwhile, the hydrogel/cell composites were fixed and observed using SEM (Figure 5c and d). As shown in the SEM images, some cells were located in the pores of the hydrogels and maintained their phenotype. These results suggested that the cells on the hydrogels had good viability. Furthermore, the cell proliferation on both layers of the hydrogels was measured using the CCK-8 assay at different time intervals (Figure 5e). The cell number increased gradually with time in each layer of the hydrogel. As the culture time increased, the number of osteoblast cells after 7 days increased by over three times compared to the number after 1 day. Although the absorbance values were slightly lower in the upper hydrogel, there was no obvious difference with the lower hydrogel. This suggested that both layers of the hydrogels were advantageous for cell viability.

### *In vivo* degradability and biocompatibility of CAN-PAC hydrogel

**Figure 6:**
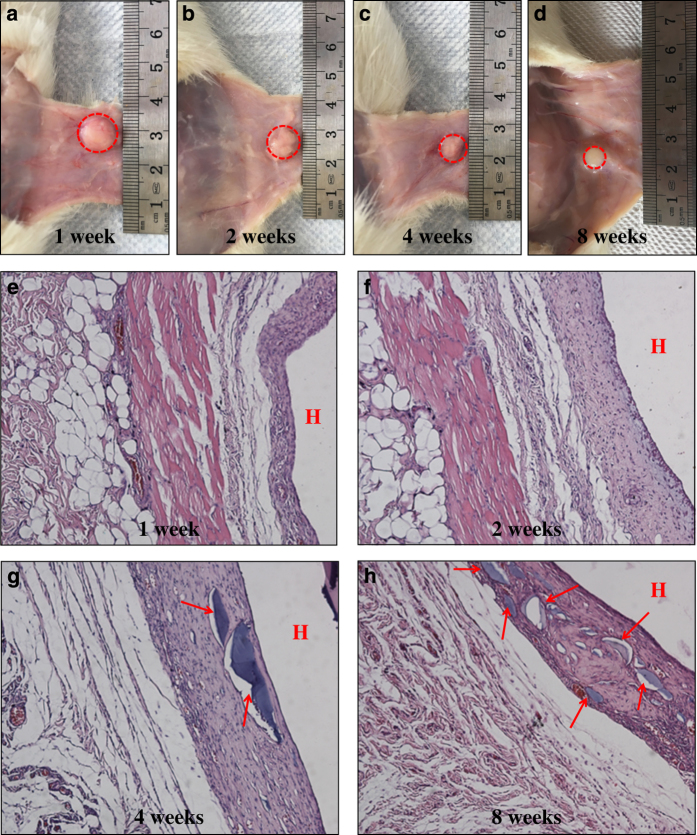
The degradability and biocompatibility of CAN-PAC hydrogel assessed in mice model. Photographs of the CAN-PAC hydrogel subcutaneously implanted in the back of rats at (**a**) 1 week, (**b**) 2 weeks, (**c**) 4 weeks and (**d**) 8 weeks (The circle indicates the tissue surrounding the CAN-PAC hydrogel). H&E staining of the tissues surrounding the implant sites at (**e**) 1 week, (**f**) 2 weeks, (**g**) 4 weeks and (**h**) 8 weeks (The red arrows and “H” denote the CAN-PAC hydrogel). The magnification was ×100.

To evaluate the *in vivo* degradation and biocompatibility of the biphasic hydrogels, hydrogels with a 1-cm diameter were subcutaneously implanted into SD rats. Over a period of two months, the surface of the hydrogel became adhered to a layer of connective tissues, and no obvious signs of infection were observed. It was observed that the hydrogels degraded gradually *in vivo* and were still integrated 4 weeks after implantation (Figure 6a–c). The hydrogels did not completely disappear after 8 weeks (Figure 6d), which indicates that the degradation time of the bilayer hydrogel was greater than 2 months.

Moreover, to assess the biocompatibility of the biphasic hydrogel *in vivo*, the tissues covering the hydrogel composites were surgically removed and stained with H&E. An increased number of inflammatory cells appeared in the connective tissue near the hydrogel during the first week (Figure 6e), suggesting an acute inflammatory response in the early period. After 2 weeks, the number of inflammatory cells had decreased, and the inflammatory response gradually disappeared (Figure 6f). Then, the tissues around the implant region had nearly returned to normal after 4 weeks (Figure 6g). In contrast, the progressive growth of host tissue into the implants implied the biocompatibility and integration of the bilayer hydrogels *in vivo* (shown by the arrows in Figure 6g and h). Therefore, the results suggest that the biodegradable bilayer hydrogel showed acceptable biocompatibility and may be suitable for *in vivo* applications.

### *In vivo* cartilage defects repair of CAN-PAC hydrogel

**Figure 7:**
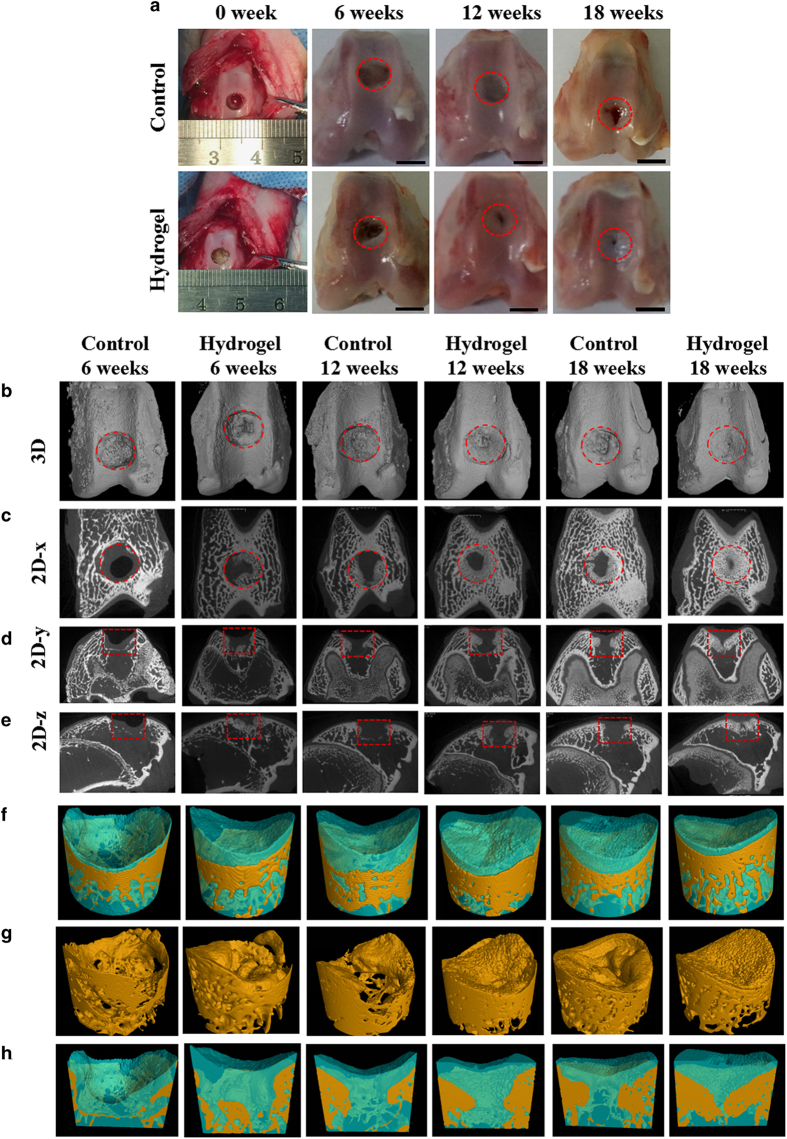

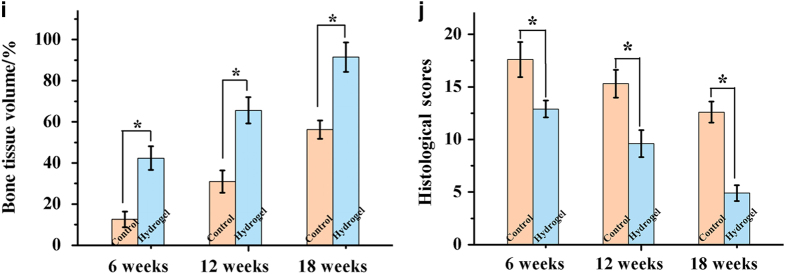
CAN-PAC hydrogel aids cartilage defect repair in rabbit model. (**a**) Photographs of knee joints in the control group and biphasic hydrogel group at the operation (0 week) and 6, 12 and 18 weeks post-operation (scale bar=5 mm). Micro-CT images obtained from (**b**) 3D reconstruction and 2D reconstruction on the (**c**) *x* axis, (**d**) *y *axis and (**e**) *z* axis of the articular joint after operation for 6, 12, and 18 weeks (red circles and red squares refer to the part of osteochondral defect). Micro-CT images isolated from the 3D reconstruction of (**f**) the repaired osteochondral defect, (**g**) the repaired subchondral bone and (**h**) the cross-section of the repaired osteochondral defect 6, 12 and 18 weeks post-operation (cartilage and bone growth are denoted in blue and yellow, respectively). (**i**) Semi-quantitative analysis of the relative amount of bone and cartilage tissues formed in the osteochondral defect model based on Micro-CT images. (**j**) Histological scoring for the repaired tissues in different groups (*P<0.05).

To test the *in vivo* efficacy of the bilayer hydrogel, osteochondral defects (5.0 mm in diameter, 4.0 mm in depth) were developed in rabbits in the center of the trochlear groove in the left knee, as shown in Figure 7a. The defects of the control group were left without treatment. Then, the capability of the bilayer hydrogel to regenerate cartilage and subchondral bone was evaluated.

#### Macroscopic observation of osteochondral repair

By gross assessment of the knee joints, no obvious inflammation and synovial hyperplasia were observed during the whole period. At 6 weeks postsurgery, the control group displayed a big hole in the defect section, and few new tissues had grown. The defects of the bilayer hydrogel group exhibited some irregular soft tissue (Figure 7a). After 12 weeks, the central region of the defects in the control group was depressed, even though some white newly grown tissue had appeared in the boundary region. On the other hand, glossy white repaired cartilage could be observed in the hydrogel group. After 18 weeks, the regenerated cartilage had grown uniformly with the original tissue, but non-repaired sections still existed in the control group. It is worthwhile to note that the defects in the hydrogel group were filled with newly formed cartilage.

#### Micro-CT analysis

Micro-CT reconstruction was used to further assess the cartilage repair. On the basis of the 3D (Figure 7b) and 2D reconstruction images from different cross-section views (Figure 7c–e) of the knee joint, it was very easy to distinguish the state of the repair. First, the neo-bone generally formed from the edge of the defects toward the central area. The bone regeneration process is similar to a bridge linking the two edges of the defect. The defects in the control group had almost not regrown and displayed an obvious gap with the adjacent tissue at 6 weeks. As the repair period progressed, the defect gradually filled with new cartilage and subchondral bone, but less void space and an irregular surface were observed. In comparison, the defects in the hydrogel group regenerated neo-cartilage and neo-subchondral bone, with only a small pinhole remaining in the central region after 18 weeks.

Furthermore, the sections of the defect in the 3D reconstruction images in the two groups were chosen as the region of interest (ROI) for analysis (Figure 7f). The regeneration of chondral and subchondral bone was easily distinguished using the denoted colors. Meanwhile, the repaired subchondral bone and cross-section of the osteochondral defect are shown in Figure 7g and h, respectively. Micro-CT analysis illustrated that the newly grown bone trabeculae of the subchondral bones gradually transitioned from loose to dense in both groups, but the control group had slower repair speed compared to the hydrogel group. Additionally, the smooth morphology of the neo-cartilage in the experiment group at 18 weeks was obvious to observe.

#### Semi-quantitative scoring analysis

The quantitative data about the new bone proportion, including cartilage and subchondral bone, could be computed from the segmented ROI (Figure 7i). The hydrogel group showed a remarkably higher score during the later period. In addition, the percentage of bone volume in the hydrogel group increased from 65.62% (12 weeks) to 91.47% (18 weeks). However, the control group had a disappointing percentage (56.23%) of new bone at 18 weeks. Then, we assessed the histological grading, as shown in Figure 7j. The scores of the group treated with hydrogels were obviously lower than those of the control group, which indicated that the bilayer hydrogel had a promising capacity to repair osteochondral defects.

#### Histological analysis of osteochondral repair

**Figure 8:**
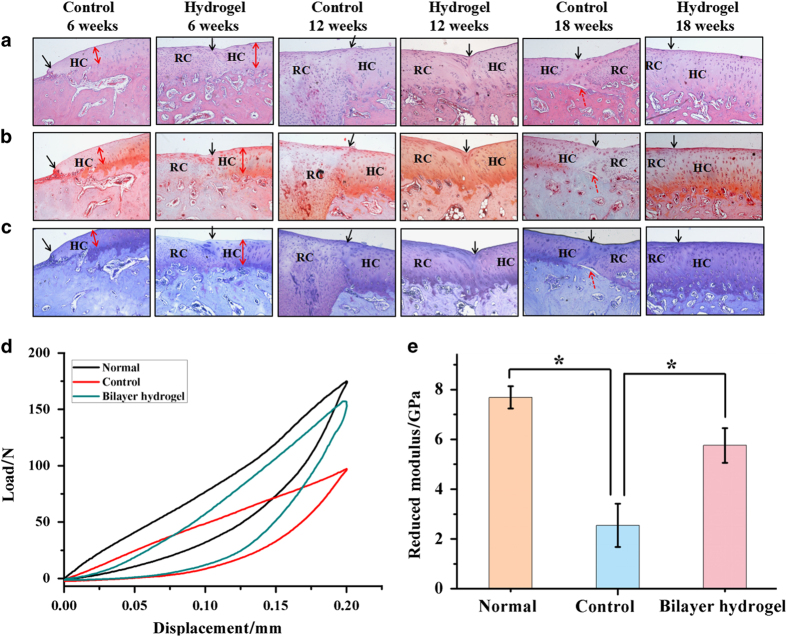
Histological staining and biomechanical property analysis of cartilage defect. (**a**) H&E staining, (**b**) safranin-O staining and (**c**) toluidine blue staining of cartilage repair in the control group and biphasic hydrogels group 6 weeks, 12 weeks and 18 weeks post-operation (the original magnification was ×100). The blank solid arrows denote the repair interface, the red solid arrows indicate the thickness of the cartilage, and the red dashed arrows represent the gap between the repaired cartilage and the subchondral bone. (**d**) Representative load-displacement curves of different groups recorded within a test range of 0.20 mm. (**e**) The reduced moduli of different groups were calculated according to the biomechanical curves. The results represent the mean±standard error of the mean; the sample number was three. *P<0.05. HC, host cartilage; RC, repaired cartilage.

Histological analysis, including H&E staining (Figure 8a), Saf-O staining (Figure 8b) and toluidine blue staining (Figure 8c), was performed. At 6 weeks after surgery, the defects treated with hydrogels showed no collapse of the adjacent tissues, and the secretion of glycosaminoglycan (GAG) in the newly grown tissue was not obvious. However, the host cartilage layer had become narrow, and the morphology of the cartilage cells displayed some changes at 6 weeks, which may contribute to the movement wear of the joint without hydrogel support. At 12 weeks, the defects in the control group exhibited disorganized and uncalcified fibrocartilage tissue in the layer of subchondral bone. On the other hand, in the biphasic hydrogel-treated group, the repaired tissue was thicker, cartilage-like tissue that showed GAG secretion. The only drawback was that the neo-cartilage was not well organized with the host cartilage. After the repair period of 18 weeks, the control group was disappointing in that the neo-cartilage layer and neo-subchondral bone layer were not integrated. These results demonstrated the limited self-regeneration ability of cartilage and the enhancement of the repair of defects by the bilayer hydrogel. Intriguingly, the regenerated cartilage tissues in the hydrogel group displayed well-organized cells perpendicular to the articular surface in clusters and columns. Meanwhile, the neo-cartilage tissue showed adequate deposition of the extracellular matrix by Saf-O and toluidine blue staining. This result indicates that our biphasic hydrogel may effectively facilitate the regeneration of cartilage defects.

#### Biomechanical property analysis

The biomechanical properties of the repaired joint samples from the two groups 18 weeks after the operation was tested. In addition, knee joint samples from normal rabbits were also evaluated. On the basis of the load-displacement curves, the cartilage repaired by the hydrogel had a significantly higher load value than the cartilage of the control group without hydrogel treatment at the same displacement (Figure 8d). As shown in Figure 8e, the reduced modulus of the repaired joint samples from the bilayer hydrogel group was ~5.758 GPa, which was 74.9% of that of normal cartilage and higher than that of the control group (3.301 GPa) (*P*<0.05). Similar to the intact cartilage in the same joint, the cartilage repaired by hydrogel system was mechanically strong enough to withstand exterior pressure. In other words, biomechanical testing suggested the CAN-PAC hydrogel had facilitated the regeneration of cartilage with similar mechanical properties as normal cartilage.

## Discussion

The osteochondral tissue is a complex structure composed of articular cartilage and subchondral bone, which have different intrinsic structures and physiological functions but integrate well with each other for weight bearing and functional joint movement.^30^ Considering that osteochondral defects involve both layers, a bilayer scaffold is needed to satisfy the requirements of osteochondral regeneration.^31,32^ In our study, the designed biomimetic bilayer CAN-PAC hydrogel had the advantage of being prepared by a one-pot heat-initiated free radical polymerization method based on different densities. The interface of the hydrogel was seamless. The upper and lower hydrogel possess different characteristics, such as morphology and mechanical and chemical features.

First, a critical factor that we need consider is the components of the scaffold, which functions as a temporary 3D template that may supply nutrient content, mechanical support and structural guidance during the tissue regeneration period.^33,34^ Furthermore, biomaterials selected for the cartilage layer and subchondral layer should have an appropriate degradation/chondrogenic induction ratio and a slow degradation/osteogenic induction ratio, respectively. Previously, robust CSMA/PECA/graphene scaffolds had been produced by our group. These scaffolds were able to promote cartilage growth and regeneration *in vitro* and *in vivo*. On the basis of these promising outcomes, we chose biologically derived CSMA as the main component for the upper layer hydrogel. To enhance the mechanical support of the lower layer, we then modified the two ends of the polymer PCEC with linked carbon-carbon double bonds to form PECDA as the main component of the lower layer hydrogel.

The bilayer CAN-PAC hydrogel was comprehensively characterized, including its morphology, pore size, porosity distribution, swelling ability and mechanical properties. The upper and lower hydrogels both presented highly interconnected porous structures. The mean pore size of the bilayer scaffold was ~147.9 μm, which may supply enough space for cell adhesion and migration during osteochondral regeneration. Additionally, the interface of the bilayer scaffold was seamless, as shown in Figure 2c. From the 2D reconstruction images obtained by Micro-CT (Figure 3c), it was shown that the bilayer hydrogel displayed a similar structure to that of cartilage and subchondral bone. The porosity distribution curve suggested that the pores were distributed uniformly in both layers and higher porosity was observed in the upper (cartilage) layer. The CAN-PAC hydrogel maintained their shape under high loading, suggesting that the binding strength of the two layers was good. The stress-strain curves (Figure 4) of the upper hydrogel and lower hydrogel from biomechanical testing showed that the hydrogel had the elasticity to support the force from joint movement.

The prepared bilayer CAN-PAC hydrogel exhibited biomimetic characteristics, which gave us the opportunity to investigate its efficacy for osteochondral defect repair. In an *in vitro* study, chondrocytes and osteoblast cells adhered and proliferated on the upper layer and lower layer, respectively. The degradability of the CAN-PAC hydrogel *in vivo* was tested by implanting it subcutaneously in rats. The hydrogel could support tissue ingrowth, displaying good biocompatibility without any obvious foreign body reaction (Figure 6g and h). The high porosity and interconnected structure may contribute to the tissue ingrowth of the bilayer hydrogel. Finally, the bilayer hydrogels were implanted in a rabbit model of osteochondral repair. The hydrogel used as a temporary structure and environment for regeneration was gradually replaced by native-like tissue. The structure and the function of the repaired tissues in hydrogel group were superior to that of the control group. The proposed CAN-PAC hydrogel could act as an effective scaffold for enhancing the regeneration of osteochondral defects. Furthermore, to investigate the mechanism of the enhanced repair by the bilayer hydrogel, further evaluation is needed. Our study provides evidence on the design of bilayer hydrogels for osteochondral regeneration. The idea behind the fabrication this biphasic hydrogel can be extended to other areas of tissue engineering,^35,36^ such as blood vessel tissue engineering and cornea tissue engineering.^37,38^

## Conclusions

In this study, we demonstrate the feasibility of preparing integrated biphasic hydrogels for full-defect cartilage regeneration using an innovative one-pot thermally reactive, rapid cross-linking method. The biphasic hydrogels displayed reasonable component and spatially controllable porosity as well as superior mechanical properties. The upper and lower hydrogels maintained the viability of cartilage cells and osteoblast cells, respectively, *in vitro*. When subcutaneously implanted in rats, the scaffolds induced a very weak inflammatory response and allowed the ingrowth of newly formed tissue, indicating that the bilayer hydrogel had appropriate biodegradability and good biocompatibility. Moreover, the cartilage regenerated *in vivo* by the hydrogel had similar structure and function to the host tissue. This approach may contribute to the clinical translation of biphasic scaffolds in cartilage tissue engineering in the future.
